# The impact of country-level structural stigma on loneliness and social capital in older and younger LGB individuals in 113 countries: Dinah Gutermuth

**DOI:** 10.1093/eurpub/ckac129.193

**Published:** 2022-10-25

**Authors:** DM Doyle, P Qualter, C Victor, M Barreto

**Affiliations:** 1 Department of Psychology, University of Exeter, Exeter, UK; 2 Manchester Institute of Education, University of Manchester, Manchester, UK; 3 Institute of Environment, Health and Societies, Brunel University, London, UK

## Abstract

**Background:**

The mental health gap between sexual minorities and heterosexuals remains a pressing issue for policy makers and scholars. In past years, numerous studies from the US found support for the vast impact structural stigma on a state level can have on the lives of LGB individuals. However, most research has been conducted within a US context and research capturing structural stigma on a country level remains scarce. In the current study we aim to close this gap by examining whether country-level structural stigma can explain loneliness and social capital among sexual minorities across the world and testing whether these relationships are different for LGB individuals from different age groups.

**Methods:**

The current study analysed a sample of over 7000 LGB people from across 113 European and non-European countries to examine the influence of country-level structural stigma on individual level loneliness and social capital.

**Results:**

Multilevel models showed that the greater structural stigma present in a country, the lower social capital was experienced by LGB respondents (b = -0.05, 95% CI: -0.07, -0.02, p < 0.001). This relationship was unaffected by respondent age. Further, multilevel models showed the following for loneliness as an outcome: The greater structural stigma present in a country, the more loneliness was experienced by LGB individuals (b = .01, 95% CI: .01, .21, p = .048). Furthermore, this relationship was moderated by respondent age (b = -.03, 95% CI: -.06, -.01, p = .01), in the sense that younger LGB people showed significantly higher levels of loneliness than older LGB people in countries with greater, but not lesser, levels of structural stigma. These effects remained robust to adjustment for demographics as well as adding country-level covariates.

**Conclusions:**

The findings of this study demonstrate the impact structural stigma on a country level can have on LGB individuals’ loneliness and social capital, differing for older and younger individuals.

